# Optimization of culture conditions to produce high yields of active *Acetobacter *sp. CCTCC M209061 cells for anti-Prelog reduction of prochiral ketones

**DOI:** 10.1186/1472-6750-11-110

**Published:** 2011-11-20

**Authors:** Xiao-Hong Chen, Wen-Yong Lou, Min-Hua Zong, Thomas J Smith

**Affiliations:** 1 Lab of Applied Biocatalysis, South China University of Technology, Guangzhou, 510640, China; 2 College of Light Industry and Food Sciences, South China University of Technology, Guangzhou, 510640, China; 3 Biomedical Research Centre, Sheffield Hallam University, Owen Building, Howard Street, Sheffield, S1 1WB, UK

## Abstract

**Background:**

Chiral alcohols are widely used in the synthesis of chiral pharmaceuticals, flavors and functional materials and appropriate whole-cell biocatalysts offer a highly enantioselective, minimally polluting route to these valuable compounds. The recently isolated strain *Acetobacter *sp. CCTCC M209061 showed exclusive anti-Prelog stereoselectivity for the reduction of prochiral ketones, but the low biomass has limited its commercialization and industrial applications. To tackle this problem, the effects of medium components and culture conditions on the strain's growth and reduction activity were explored.

**Results:**

By using a one-at-a-time method and a central composite rotatable design (CCRD), the optimal medium and culture conditions were found to be as follows: glucose 8.26 g/L, fructose 2.50 g/L, soy peptone 83.92 g/L, MnSO_4_·H_2_O 0.088 g/L, pH 5.70, 30°C and 10% (v/v) inoculum. Under the above-mentioned conditions, the biomass after 30 h cultivation reached 1.10 ± 0.03 g/L, which was 9.5-fold higher than that obtained with basic medium. Also, the reduction activity towards 4'-chloroacetophenone was markedly enhanced to 39.49 ± 0.96 *μ*mol/min/g from 29.34 ± 0.65 *μ*mol/min/g, with the product *e.e*. being above 99%. Comparable improvements were also seen with the enantioselective bioreduction of 4-(trimethylsilyl)-3-butyn-2-one to the key pharmaceutical precursor (*R*) - 4-(trimethylsilyl)-3-butyn-2-ol.

**Conclusions:**

The biomass and reduction activity of *Acetobacter *sp. CCTCC M209061 can be greatly enhanced through the optimization strategy. This facilitates use of the strain in the anti-Prelog stereoselective reduction of prochiral ketones to enantiopure chiral alcohols as building blocks for many industries.

## Background

*Acetobacter *is a genus of acetic acid bacteria characterized by the ability to convert ethanol to acetic acid in the presence of oxygen. *Acetobacter *sp. is widely used in various fields of biotechnology [1]. The longest-established applications of this genus are the strains used for producing vinegar by oxidation of ethanol to acetic acid [2]. In addition, bacterial cellulose produced by *Acetobacter xylinum *has attracted attention because of its unique physical and mechanical properties such as its existence as pure cellulose aggregate, high crystallinity, high biocompatibility, and hence its promising properties for application in composite membranes [3], medical materials [4], electronic paper [5] and fuel cells [6]. Strains of *Acetobacter *have also proven to be efficient biocatalysts for the enantioselective oxidation of various alcohols to yield optically pure carboxylic acids. For example, Molinari et al. studied the oxidation of 2-phenylpropanol with *Acetobacter aceti *[7], prochiral 2-methyl-1, 3-propandiol by *Acetobacter pasteurianus *[8], and polyconjugated compounds catalyzed by *Acetobacter aceti *[9]. However, there have been only a few studies of the application of *Acetobacter *for asymmetric reduction of prochiral ketones. A novel strain, *Acetobacter *sp. CCTCC M209061, reported in our previous paper [10], isolated from Chinese kefir grains, showed exclusive anti-Prelog stereoselectivity for the reduction of 4-(trimethylsilyl)-3-butyn-2-one to (*R*)-4-(trimethylsilyl)-3-butyn-2-ol, which is a key chiral intermediate for the synthesis of (*R*)-benzyl-4-hydroxyl-2-pentynoate, a molecule that has potential therapeutic function for Alzheimer's disease. Also, *Acetobacter *sp. CCTCC M209061 effectively catalyzed asymmetric reduction of a series of prochiral aryl ketones and hence may be suitable for synthesis of valuable chiral alcohols. Whilst the substrate range and enantioselectivity of *Acetobacter *sp. CCTCC M209061 are promising and have enabled a biotransformation that has not been effectively accomplished with other strains, the low biomass density produced by cultures of this strain is a limitation for its application and commercialization. For example, the twelve strains of acetic acid bacteria investigated previously by Romano et al. for use in enantioselective biocatalysis gave average culture biomass densities between 1.0 to 4.4 g/L(dry weight [dw]) [11]. In contrast, *Acetobacter *sp. CCTCC M209061 yielded much less biomass in basic medium (0.11 g/L dw) and tomato juice medium (0.34 g/L dw). Previous reports in the literature have shown that culture conditions can greatly influence alcohol dehydrogenase (ADH) activities from acetic acid bacteria such as *Acetobacter *sp. [12] and *Gluconobacter oxydans *[13]. Thus, the current study was undertaken with the aim of more thoroughly exploring the growth medium composition and culture conditions to improve growth and reduction activity of the novel strain *Acetobacter *sp. CCTCC M209061 toward practical applications.

Whilst the classical 'one-factor-at-a-time' method has the severe limitation that it does not allow investigation of interactions between variables, it can be useful under certain circumstances, such as when the primary goal is to attain improvements in performance (rather than necessarily finding the global maximum) and where experimental error is small compared to the effect of the factors under study [14]. Among multivariate optimization methods that can be used more reliably to find global maxima, the response surface methodology (RSM) has previously been shown to be powerful and practicable for program optimization and is often used to identify the relative significance of different factors, interactions between factors and optimal level of test variables. For example, the RSM method was successfully applied to enhance mycelial biomass and polysaccharides produced by *Hericium erinaceum *[15], biosurfactant produced by *Bacillus mycoides *[16], protease produced by *Bacillus clause *[17] and avermectin B1a produced by *Streptomyces avermitilis *14-12A [18].

In the present work, the important influential factors affecting the growth and activity of the new strain were initially unknown and so there were a potentially very large number of permutations to study if a multivariate optimization method such as RSM were used from the outset. We therefore began by using a systematic one-factor-at-a-time optimization method to investigate the effects of medium components and culture conditions on the growth and reduction activity of *Acetobacter *sp. CCTCC M209061. The response surface methodology was then employed, in order to further improve the biomass and the specific activity of the novel strain by more rigorous exploration of the conditions that were indicated as important by the one-factor-at-a-time results. The biocatalytic asymmetric reduction of 4'-chloroacetophenone to (*R*)-1-(4-chlorophenyl) ethanol was employed as a model reaction for evaluating the effects of the various culture condition on the activity of the strain. In addition, the enantioselective reduction of 4-(trimethylsilyl)-3-butyn-2-one to (*R*)-4-(tri-methylsilyl)-3-butyn-2-ol catalyzed by cells in improved media was investigated.

## Results and discussion

### Effect of the carbon source

**Figure 1:**
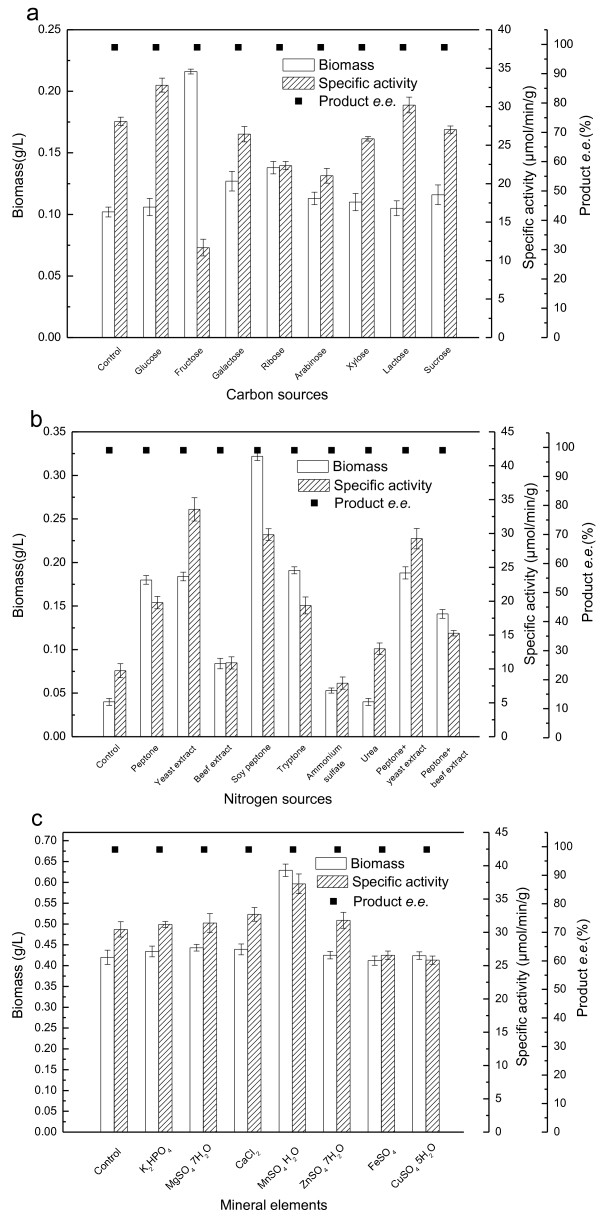
**Effect of medium components on cell growth and reduction activity of *Acetobacter *sp. CCTCC M209061**. (a) Effect of carbon sources; (b) Effect of nitrogen sources; (c) Effect of mineral elements. All experimental data are given as the mean ± SD of triplicate determinations. Control refers to the medium with no added carbon source (a), nitrogen source (b), or mineral element (c) added. The substantial growth observed in the no carbon source control in panel (a) results from the presence of glucose in the seed medium and nitrogen sources (yeast extract and peptone), which can provide a certain amount of carbon. The mixed nitrogen sources investigated in panel (b) contained equal masses of each of two nitrogen sources stated.

During the growth of microorganisms, the carbon source in the culture medium plays an important role for the growth of cells, the production of metabolites and the availability of energy to drive endergonic reactions. According to previous studies, the source of carbon for *Acetobacter *is species and strain dependent. To establish the effect of carbon sources on the growth and reduction activity of *Acetobacter *sp. CCTCC M209061, the carbohydrates glucose, fructose, galactose, arabinose, ribose, xylose, lactose and sucrose were tested individually as carbon sources (at 10 g/L) in place of the glucose in the basic medium; the control medium was the basic medium with the omission of glucose. The results (Figure 1a) showed that fructose-containing medium gave most abundant cell growth, but lowest reduction activity. In contrast, the glucose-containing medium gave highest specific reduction activity, but relatively poor cell growth. These contrasting effects of carbon source on biomass yield and enzyme activity were consistent with previous studies on *Acetobacter *strains. In *Acetobacter methanolicus *and *Acetobacter aceti *it was found previously that carbon sources which were advantageous to growth did not give best activities of alcohol dehydrogenase (ADH), owing in part to the formation of an inactive form of ADH during growth in certain media [12]. Moreover, addition of glucose influenced the activities of individual components of the electron-transport systems of the aerobic bacteria *Azotobacter vinelandii *and *Acetobacter suboxydans *[19], which suggested that carbon source may influence the availability of electron donors for biocatalytic reduction reactions. Since fructose gave best biomass yield and glucose gave best specific reduction activity, media containing mixtures of fructose and glucose were investigated (with fructose: glucose ratios of 10:0; 7.5:2.5; 5:5; 2.5:7.5 and 0:10, by weight; the total carbon source concentrations were kept at 10 g/L). It was found that the medium containing 7.5 g/L glucose and 2.5 g/L fructose gave the best combination of cell growth (0.20 ± 0.01 g/L) and reduction activity (29.14 ± 0.83 *μ*mol/min/g).

### Effect of nitrogen source

Various organic and inorganic nitrogen sources (2.15 g/L calculated as nitrogen) were also examined for their effects on growth and reduction activity in place of the peptone and yeast extract in the basic medium (Figure 1b). Cells grown in the medium containing soy peptone as nitrogen source produced the largest amount of biomass. Indeed, soy peptone is a complex ingredient which contains several essential amino acids as well as many other growth-promoting factors and it is known that its addition can have a positive influence on biomass production [20]. Lower biomasses were observed in culture media containing other complex nitrogen sources (peptone, yeast extract and tryptone) and the *Acetobacter *sp. CCTCC M209061 grew very little in media with ammonium sulfate or urea as the nitrogen source, or in the control medium without the addition of a nitrogen source.

Of all the nitrogen sources tested, the highest reduction activity was obtained with yeast extract. High reduction activities were also obtained in media with soy peptone and a mixture of peptone and yeast extract. The other organic and inorganic nitrogen sources gave substantially lower reduction activities and were judged unsuitable for producing highly active biocatalyst. Mixtures of soy peptone and yeast extract (the ratios were 1:0; 3:1; 1:1; 1:3 and 0:1, total nitrogen concentration held at 2.15 g/L) were also tested as nitrogen source, but the result was no better than soy peptone alone (26.9 g/L), therefore, soy peptone (which gave the best combination of biomass yield and specific reduction activity) was chosen as the nitrogen source for subsequent experiments. The effects of different concentrations of soy peptone (40-80 g/L) were also investigated. Biomass and specific activity were improved with the increase of concentration, but higher than 70 g/L gave negligible improvement and so a concentration of soy peptone of 70 g/L was selected for further study which gave a high cell growth (0.32 ± 0.01 g/L) and reduction activity (29.83 ± 0.87 *μ*mol/min/g).

### Effect of mineral element

Seven mineral elements (potassium, magnesium, calcium, zinc, manganese, ferrous and copper ions) were chosen for investigation as medium additives based on their known roles in bacterial metabolism and as cofactors for metabolic enzymes [21,22]. Addition of the various mineral elements to the culture medium was investigated by adding the salts K_2_HPO_4_, MgSO_4_·7H_2_O, CaCl_2_, MnSO_4_·H_2_O, ZnSO_4_·7H_2_O, FeSO_4 _and CuSO_4_·5H_2_O to separate aliquots of media to concentrations of 2.0, 1.0, 1.0, 0.05, 0.02, 0.003 and 0.005 g/L, respectively, in place of the K_2_HPO_4 _in the basic medium. These concentrations were selected based on the requirement for these elements for the growth of microorganisms in previous studies, and to keep their concentrations below expected inhibitory levels [23]. Most of the minerals did not have a substantial effect on yields of biomass or reduction activity (Figure 1c); however, Mn^2+ ^improved the growth and reduction activity by extending the logarithmic growth phase. It has been reported previously that manganese is an important component of tomato juice when used for the cultivation of microorganisms [24], which was consistent with our result and suggested that supplementation with manganese may be an alternative to addition of tomato juice to the medium for cultivation of *Acetobacter *sp. CCTCC M209061. The addition of K, Mg, Ca and Zn ions has been found to improve cell growth and product yields in other studies [25,15] but afforded negligible benefits here, presumably because none of these minerals was limiting for cell growth or reduction activity. Fe and Cu ions slightly inhibited reduction activity, which may be due to their relatively high concentrations for this strain. To determine the effect of Mn^2+ ^concentration on the biomass and reduction activity of *Acetobacter *sp. CCTCC M209061, different concentrations of MnSO_4_·H_2_O ranging from 0 to 0.10 g/L were added to the medium. A manganese concentration of 0.075 g/L gave a maximum biomass yield of 0.94 ± 0.04 g/L and a maximum specific reduction activity of 34.15 ± 1.05 *μ*mol/min/g.

### Effects of extra vitamins

Vitamins play a key role for many microorganisms because they act as coenzymes or precursors of coenzymes [26]; they are therefore often crucial for cell growth and enzyme activities at very small concentrations. Indeed, complex nutrient sources such as soy peptone contain certain vitamins at small concentrations, so it is possible that part of the beneficial effect of adding such preparations to the medium was because they were supplying vitamin(s). To investigate the effect of vitamins specifically, the following vitamins were added to the medium: thiamine (vitamin B1), nicotinic acid (vitamin B3), pyridoxine (vitamin B6) and ascorbic acid (vitamin C), biotin (vitamin H or B7), each at a concentration of 2.0 mg/L. However, none of the vitamins examined produced notable differences from the control without added vitamins (Table 1). The reason may be that such vitamins that the culture required were already present in the soy peptone component of the medium.

**Table 1:** Effects of culture factors on cell growth and reduction activity of *Acetobacter *sp. CCTCC M209061


**Factor**	**Biomass (g/L)**	**Specific activity (*μ*mol/min/g)**	***e.e*. (%)**

Extra vitamins			

Control	0.94 ± 0.04	34.15 ± 1.05	≥ 99
Vitamin B1	0.93 ± 0.03	34.36 ± 0.94	≥ 99
Nicotinic acid	0.93 ± 0.01	35.04 ± 1.07	≥ 99
Pyridoxine	0.95 ± 0.02	34.18 ± 0.89	≥ 99
Ascorbic acid	0.94 ± 0.03	33.88 ± 0.93	≥ 99
Biotin	0.93 ± 0.04	34.59 ± 0.86	≥ 99

pH			

4	0.38 ± 0.01	11.61 ± 0.39	≥ 99
5	0.95 ± 0.02	34.67 ± 1.10	≥ 99
6	0.94 ± 0.04	34.15 ± 1.05	≥ 99
7	0.82 ± 0.03	29.37 ± 0.84	≥ 99
8	0.48 ± 0.01	23.03 ± 0.58	≥ 99

Temperature (°C)			

25	0.79 ± 0.03	33.01 ± 1.10	≥ 99
30	0.95 ± 0.02	34.67 ± 1.10	≥ 99
35	0.86 ± 0.02	31.38 ± 1.05	≥ 99
40	0.62 ± 0.02	23.21 ± 0.61	≥ 99
45	0.08 ± 0.01	0	-

Shaking rate (rpm)			

40	0.76 ± 0.02	39.55 ± 1.12	≥ 99
80	0.96 ± 0.02	36.76 ± 1.03	≥ 99
100	0.95 ± 0.02	34.67 ± 1.10	≥ 99
120	0.97 ± 0.02	30.14 ± 0.99	≥ 99
160	0.96 ± 0.03	24.24 ± 0.65	≥ 99
200	0.97 ± 0.04	17.74 ± 0.47	≥ 99

### Effects of the initial pH

The pH value of culture medium can affect the functions of the cell membrane, the cell structure, the uptake of various nutritional sources, and the biosynthesis of metabolites [27]. The effects of the initial pH of the culture medium on growth and reduction activity of *Acetobacter *sp. CCTCC M209061 were studied. The initial pH 5 gave the best combination of cell growth (0.95 ± 0.02 g/L) and reduction activity (34.67 ± 1.10 *μ*mol/min/g). Cell growth and reduction activity were inhibited as the pH increased above 6 and no growth was observed when the initial pH was ≥ 9 or ≤3 (Table 1). It can be seen that the growth and reduction activity changed very little between pH 5 and 6, which confirmed a well-established observation that *Acetobacter *sp. grow best under acidic conditions [28].

### Effects of temperature and shaking rate

The effects of different culture temperatures and shaking rate were also investigated; the optimum growth and reduction activity were gained at 30°C and 80 rpm (Table 1). The biomass yield and specific reduction activity were 0.96 ± 0.02 g/L and 36.76 ± 1.03 *μ*mol/min/g respectively at the conditions optimized by the one-at-a-time method.

### Optimization by response surface methodology

As detailed above, RSM is a useful and practicable method among different multivariate optimization methods. Based on the results obtained from the one-factor-at-a-time experiments for optimizing the culture medium, the four variables that exhibited greatest effects on cell growth and reduction activity were chosen for CCRD. These were: carbon source, nitrogen source, mineral element and the initial pH. Glucose (supplemented with a fixed concentration of 2.5 g/L of fructose in the medium), soy peptone and Mn^2+ ^were selected as the medium components (carbon source, nitrogen source and mineral element, respectively) for further optimization based on the fact that these gave the best reduction activities and biomass yields during the one-at-a-time optimization. In the CCRD experiment the interactions of these medium components were investigated, together with the medium pH.

Table 2 showed the design matrix for the CCRD experiment, together with the experimental results and the predicted responses for growth and reduction activity of *Acetobacter *sp. CCTCC M209061. The experimental values obtained from the CCRD were regressed by using a quadratic polynomial equation, and the two regression equations, expressed in terms of the coded factors defined in Table 3 were as follows:

**Table 2:** The design and results of the central composite rotatable design (CCRD) experiments


**Run**	**Factor**				**Biomass (g/L)**		**Specific activity (*μ*mol/min/g)**	
			
	**X_1_**	**X_2_**	**X_3_**	**X_4_**	**Observed**	**Predicted**	**Observed**	**Predicted**

1	-1	-1	-1	-1	1.02	1.03	36.35	36.42
2	1	-1	-1	-1	1.13	1.13	33.61	33.52
3	-1	1	-1	-1	0.96	0.95	40.03	40.02
4	1	1	-1	-1	1.15	1.14	35.43	35.36
5	-1	-1	1	-1	1.01	1.00	32.87	33.03
6	1	-1	1	-1	1.03	1.03	33.12	33.02
7	-1	1	1	-1	0.97	0.96	35.56	35.59
8	1	1	1	-1	1.07	1.07	34.01	33.82
9	-1	-1	-1	1	0.92	0.93	38.35	38.36
10	1	-1	-1	1	1.04	1.05	36.15	35.99
11	-1	1	-1	1	0.85	0.85	41.35	41.31
12	1	1	-1	1	1.05	1.06	37.50	37.17
13	-1	-1	1	1	0.88	0.88	40.23	40.17
14	1	-1	1	1	0.91	0.92	40.86	40.69
15	-1	1	1	1	0.82	0.82	42.16	42.07
16	1	1	1	1	0.97	0.96	41.04	40.83
17^a^	-2	0	0	0	0.83	0.83	38.43	38.24
18^a^	2	0	0	0	1.07	1.07	33.59	34.10
19^a^	0	-2	0	0	1.03	1.01	36.76	36.77
20^a^	0	2	0	0	0.96	0.97	40.21	40.51
21^a^	0	0	-2	0	1.06	1.05	35.09	35.25
22^a^	0	0	2	0	0.91	0.92	35.36	35.52
23^a^	0	0	0	-2	1.11	1.12	34.73	34.67
24^a^	0	0	0	2	0.93	0.91	43.26	43.63
25^b^	0	0	0	0	1.04	1.06	40.32	39.77
26^b^	0	0	0	0	1.05	1.06	39.07	39.77
27^b^	0	0	0	0	1.06	1.06	39.44	39.77
28^b^	0	0	0	0	1.07	1.06	40.23	39.77
29^b^	0	0	0	0	1.05	1.06	39.19	39.77
30^b^	0	0	0	0	1.07	1.06	40.37	39.77

X_1_, glucose; X_2_, soy peptone; X_3_, MnSO_4_·H_2_O; X_4_, initial pH.

^a ^Axial point.

^b ^Central point.

**Table 3:** Factors and levels in central composite rotatable design (CCRD)


**Factor**	**Name**	**Coded level**				
		
		-2	-1	0	1	2

X_1_	Glucose (g/L)	2.5	5	7.5	10	12.5
X_2_	Soy peptone (g/L)	60	70	80	90	100
X_3_	MnSO_4_·H_2_O (g/L)	0.07	0.08	0.09	0.10	0.11
X_4_	pH	4.5	5	5.5	6	6.5

(1) Y1=1.06+0.058X1-0.010X2-0.031X3-0.052X4+0.022X1 X2-0.020X1 X3+0.0053X1 X4+0.0059X2 X3-0.0016X2 X4-0.0064X3 X4-0.026X12-0.016X22-0.018X32-0.0099X42

(2) Y2=39.77-1.04X1+0.94X2+0.067X3+2.24X4-0.44X1 X2+0.72X1 X3+0.13X1 X4-0.26X2 X3-0.16X2 X4+1.30X3 X4-0.90X12-0.28X22-1.10X32-0.15X42

where *Y*_1 _is the biomass (g/L), *Y*_2 _is the reduction activity (*μ*mol/min/g), and *X*_1_, *X*_2_, *X*_3 _and *X*_4 _are coded values of the independent variables (glucose; soy peptone; MnSO_4_·H_2_O and pH). The other culture conditions were as fixed as follows, based on the results from the one-at-a-time optimization: temperature 30°C, shaking rate 80 rpm, inoculum 10% (v/v), cultivation time 30 h.

The analysis of variance (ANOVA) for the CCRD experiments was performed (Table 4). The *F*-values and *P*-values were used to identify the effect of each factor on biomass and reduction activity. The models exhibited remarkable correlation with the experimental data with very high *F*-values (73.18 and 101.91 for biomass and reduction activity, respectively) and very low *P *< 0.0001 for each model, which implied the significance of the two models (*P*-values less than 0.05 are generally taken to indicate that model terms are significant). By inspection of the *F*- and *P*-values of each factor, it can be seen that glucose had the largest effect on biomass, followed by pH, MnSO_4_·H_2_O and soy peptone. The order of effects on reduction activity was pH > glucose > soy peptone. Within the examined concentration range (0.07-0.11 g/L), change of MnSO_4_·H_2_O concentration showed only slight impact on the reduction activity. It is noteworthy that the variable that had greatest influence on the production of biomass (glucose concentration) was different from that which exerted most significant impact on the reduction activity (pH of the medium). It is well known that the metabolic networks of microorganisms are very complicated and respond in a complex manner to environmental conditions. Here, the balance of these factors clearly resulted in a different response of reduction activity and cellular growth to the various culture conditions explored. It is possible that glucose concentration had greatest effect upon accumulation of biomass because cellular growth was limited to a large extent by the availability of glucose as the principal carbon and energy source of the cells. whilst there were many possible explanations of the large influence of medium pH on the reduction activity towards ketone substrates, including direct effects on enzyme activity and indirect ones mediated via changes in gene expression and the availability of electron donors. It is reasonable that the conditions that best favor growth are different from those that give highest reduction activity because the two processes may compete for the pool of available electron donors within the cell.

**Table 4:** Analysis of variance (ANOVA) for the experimental results of the CCRD


**Source**	**Biomass (g/L)**					**Specific activity (*μ*mol/min/g)**				
	
	**SS**	**df**	**SM**	***F*-value**	*P*-value*	**SS**	**df**	**SM**	***F*-value**	*P*-value*

Model	0.22	14	0.015	73.18	< 0.0001	256.26	14	18.30	101.9	< 0.0001
X_1_	0.081	1	0.081	384.8	< 0.0001	25.74	1	25.74	143.3	< 0.0001
X_2_	0.0024	1	0.0024	11.30	0.0043	20.99	1	20.99	116.8	< 0.0001
X_3_	0.024	1	0.024	112.7	< 0.0001	0.11	1	0.11	0.6066	0.4482
X_4_	0.065	1	0.065	307.5	< 0.0001	120.26	1	120.26	669.5	< 0.0001
X_1_X_2_	0.0079	1	0.0079	37.38	< 0.0001	3.11	1	3.11	17.33	0.0008
X_1_X_3_	0.0062	1	0.0062	29.43	< 0.0001	8.40	1	8.40	46.80	< 0.0001
X_1_X_4_	0.00045	1	0.00045	2.143	0.1638	0.28	1	0.28	1.539	0.2338
X_2_X_3_	0.00056	1	0.00056	2.677	0.1226	1.08	1	1.08	6.031	0.0267
X_2_X_4_	0.00004	1	0.00004	0.1850	0.6729	0.43	1	0.43	2.383	0.1435
X_3_X_4_	0.00066	1	0.00066	3.147	0.0964	27.03	1	27.03	150.5	< 0.0001
X_1_^2^	0.019	1	0.019	91.35	< 0.0001	22.26	1	22.26	123.9	< 0.0001
X_2_^2^	0.0068	1	0.0068	32.25	< 0.0001	2.18	1	2.18	12.15	0.0033
X_3_^2^	0.0088	1	0.0088	41.55	< 0.0001	33.01	1	33.01	183.8	< 0.0001
X_4_^2^	0.0027	1	0.0027	12.67	0.0029	0.68	1	0.66	3.648	0.0755
Lack of fit	0.0025	10	0.00025	1.89	0.2497	0.88	10	0.088	0.24	0.9722
R^2 ^= 0.9856						R^2 ^= 0.9896				

X_1_, glucose; X_2_, soy peptone; X_3_, MnSO_4_·H_2_O; X_4_, initial pH.

*P < 0.05 are significant.

SS: Sum of squares; DF: degrees of freedom; SM: mean squares.

The closer the value of *R*^2 ^is to 1, the better is the adequacy of the model. In this case, the *R*^2 ^values (0.9856 and 0.9896) indicated that actual values were very close to predicted values and showed that the models were reliable for predicting biomass and reduction activity, respectively, of *Acetobacter *sp. CCTCC M209061. The "Lack of Fit *F*-value" which were 1.89 (*P*-value 0.25 > 0.05) and 0.24 (*P*-value 0.97 > 0.05) also implied that the predicted values exhibit a good correlation with the experimental data. Hence, all of the parameters indicated that the model was adequate for prediction.

**Figure 2:**
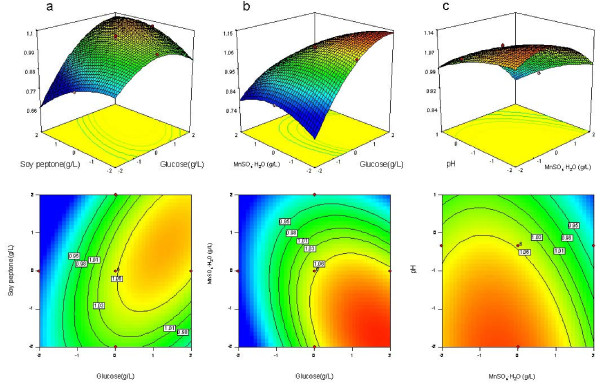
**Response surfaces and corresponding contour plots obtained from Equation (1)**. The combined effects of (a) glucose (X_1_) and soy peptone (X_2_); (b) glucose (X_1_) and MnSO_4_·H_2_O (X_3_); (c) MnSO_4_·H_2_O (X_3_) and initial pH (X_4_) on growth of *Acetobacter *sp. CCTCC M209061.

**Figure 3:**
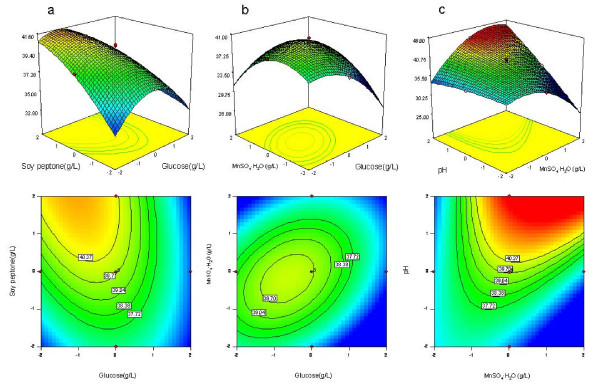
**Response surfaces and corresponding contour plots obtained from Equation (2)**. The combined effects of (a) glucose (X_1_) and soy peptone (X_2_); (b) glucose (X_1_) and MnSO_4_·H_2_O (X_3_); (c) MnSO_4_·H_2_O (X_3_) and initial pH (X_4_) on reduction activity of *Acetobacter *sp. CCTCC M209061.

Three-dimensional response surface plots and corresponding contour plots were constructed to show the effects of the conditions on growth (Figure 2) and reduction activity (Figure 3) of *Acetobacter *sp. CCTCC M209061. The effects of independent variables are shown in pairs within the experimental range, while the other two variables in each case are fixed at center point levels. This kind of graphical visualization allows the relationships between the experimental levels of each factor and the response to be investigated, and the type of interactions between test variables to be determined, which is necessary to establish the optimal medium components and culture conditions. The elliptical nature of the curves indicates significant mutual interactions between variables, in contrast to the circular shapes of the contour plots. There was a large interactive effect of glucose and soy peptone on growth of *Acetobacter *sp. CCTCC M209061 (Figure 2a). At low to moderate glucose concentration, the biomass increased marginally with the increase of soy peptone concentration, but at high glucose concentration, increase in soy peptone concentration led to much greater increase of biomass. Substantial interaction was also observed between the effects on cell growth of the other pairs of variables shown, namely MnSO_4_·H_2_O and pH (Figure 2b) and glucose and MnSO_4 _·H_2_O (Figure 2c).

When specific activity was considered, the strongest interaction between variables was that between MnSO_4_·H_2_O and pH (Figure 3b), followed by the interaction between glucose and soy peptone (Figure 3a) and that between soy peptone and pH (Figure 3c).

The optimum levels of the factors investigated can be deduced from the 3D and 2D response surface plots and the equations obtained from multiple regression analysis. The model predicted that the maximum cell growth (1.17 g/L) was located at X_1 _= 11.89 g/L, X_2 _= 87.44 g/L, X_3 _= 0.076 g/L, and X_4 _= 4.62. Likewise, the predicted specific activity (45.34 *μ*mol/min/g) reached its maximum at the values: X_1 _= 7.07 g/L, X_2 _= 87.16 g/L, X_3 _= 0.10 g/L, and X_4 _= 6.5. The predicted levels of factors to maximize biomass were rather different from those predicted to maximize the specific reduction activity. Although specific activity requires moderate concentration of glucose and relative high pH, maximal biomass was achieved in the presence of a relative high concentration of glucose and low pH. For instance, in the nutrient medium needed for maximal specific activity (45.34 *μ*mol/min/g), the predicted biomass was 0.82 g/L, which was not satisfactory for practical applications. Thus, numerical optimization of the overall desirability function was performed to determine the best possible goals for each response simultaneously. The predicted optimal values for the variables were as follows: X_1 _= 8.26 g/L, X_2 _= 83.92 g/L, X_3 _= 0.088 g/L, and X_4 _= 5.70, and the predicted responses were: biomass, 1.05 g/L; specific activity 40.37 *μ*mol/min/g.

To examine the validity of this model, nine successive experiments were performed in the predicted optimal medium. The average biomass (1.10 ± 0.03 g/L) and specific activity (39.49 ± 0.96 *μ*mol/min/g) were very close to the predicted values and strongly support the suitability of the CCRD model developed in this study for improving the reaction.

### Comparison of cell growth and reduction activity of the strain in the improved and original basic media

**Figure 4:**
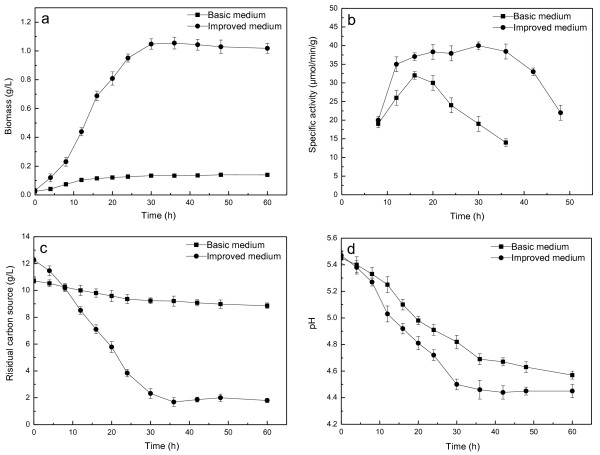
**Comparison of growth and reduction activity of *Acetobacter *sp. CCTCC M209061 in various medium**.

The improved (optimized) medium described in the previous section was compared for growth and reduction activity of *Acetobacter *sp. CCTCC M209061 to the original basic medium used at the start of the study (Figure 4). Cells cultured in the improved medium had longer logarithmic phase and reached the maximum of 1.10 ± 0.03 g/L DCW at 30 h. The biomass yield was 9.5 times higher than that from the basic medium. Reduction activity reached 39.49 ± 0.96 *μ*mol/min/g in the improved medium, which was 1.33-fold higher than that in basic medium (29.34 ± 0.65 *μ*mol/min/g). Hence, the optimization described in this study increased the reduction activity per litre of culture by more than 12-fold. The reduction activity declined sharply after reaching its maximum value in basic medium, however, in the improved medium cells maintained near-maximum activity between 12 and 36 h after inoculation. The reason for the improved stability of the activity may be that the presence of added manganese allows the reduction activity to be maintained for longer. The carbon source conversion in the improved medium was higher than in the basic medium (Figure 4c), which was consistent with the biomass yield results and suggested that a greater amount of carbon source was available for growth and reduction activity by *Acetobacter *sp. CCTCC M209061 in the improved medium. As shown in Figure 4d, the pH decreased during cultivation in both media, but fell significantly more in the improved medium.

### Performance of the optimized biocatalyst for enantioselective reduction of 4-(trimethylsilyl)-3-butyn-2-one

Whilst the increase in biocatalyst biomass due to the optimization of the culture medium would benefit any reduction reaction catalyzed by *Acetobacter *sp. CCTCC M209061, it was possible that the change in culture conditions might affect the rate of reduction of different substrates to different extents. The cells cultured in the improved medium were therefore tested as the biocatalyst for the key enantioselective reduction of 4-(trimethylsilyl)-3-butyn-2-one to (*R*) - 4-(trimethylsilyl)-3-butyn-2-ol that we investigated previously [10]. Compared with the previous work, using cells prepared from the optimized reaction medium, under the reduction reaction conditions described previously, the initial reaction rate increased from 0.5 *μ*mol/min to 1.13 *μ*mol/min; the yield was enhanced from 71% to 83% and the product *e.e*. was > 99% after a reaction time of 40 min. Hence the combined effects of increased biocatalyst biomass and increased yield would afford an extensive increase in the productivity of this reaction, which was comparable to the improvement achieved with the model reduction reaction used for the optimization. These results also represented an enantiocomplementary transformation to the reduction of 4-(trimethylsilyl)-3-butyn-2-one to (*S*) - 4-(trimethylsilyl)-3-butyn-2-ol catalysed by immobilized *Candida parapsilosis *CCTCC M203011 cells in an ionic liquid system [29].

## Conclusions

Cultivation conditions were optimized for *Acetobacter *sp. CCTCC M209061, which acted as an anti-Prelog specific whole cell biocatalysts for reduction of prochiral ketones. Using a classical one-factor-at-one-time method followed by a response surface methodology, culture conditions were optimized to obtain simultaneously high yields of biomass (1.10 g/L) and high reduction activity (39.49 *μ*mol/min/g with the model substrate 4'-chloroacetophenone). These results, which gave a more than 12-fold improvement in the yield of reduction activity per litre of culture, were expected to facilitate use of *Acetobacter *sp. CCTCC M209061 cells in the production of chiral alcohols as building blocks for many industries.

## Methods

### Biological and chemical materials

The new strain, *Acetobacter *sp. CCTCC M209061, was isolated previously from Chinese kefir grains and kept in our laboratory [10].

4'-Chloroacetophenone (97% purity), (*R*)-1-(4-chlorophenyl) ethanol (> 95% purity), 1-(4-chlorophenyl) ethanol (> 98% purity), 3'-methoxyacetophenone (97% purity), 4-(trimethylsilyl)-3-butyn-2-one (97% purity), 4-(tri-methylsilyl)-3-butyn-2-ol (97% purity) and n-decane (> 99% purity) were purchased from Sigma-Aldrich (USA). All other chemicals were from commercial sources and were of analytical grade.

### Cultivation of Acetobacter sp. CCTCC M209061

Seed medium (tomato juice medium) consisted of 10 g/L glucose, 10 g/L peptone, 10 g/L yeast extract, 20% (v/v) tomato juice and was adjusted to pH 6.0 [10]. Basic medium, which was used as the starting point for optimization of the culture conditions, contained 10 g/L glucose as the carbon source, 10 g/L peptone and 10 g/L yeast extract as the nitrogen source, and a mineral element component in the form of 2 g/L of K_2_HPO_4_; pH was adjusted to 6.0. The media were sterilized by autoclaving at 121°C for 20 min.

Initial cultivation of *Acetobacter *sp. CCTCC M209061 was performed in 250 mL Erlenmeyer flasks containing 45 mL of basic medium, which was inoculated by adding 5 mL of starter culture in tomato juice medium for 13 h cultivation. The culture was grown at 30°C and a shaking rate of 120 rpm. Wet cells were harvested at the late exponential growth phase (20 h without Mn^2+ ^and 30 h with Mn^2+^) by centrifugation (4°C, 8000 rpm for 10 min), washed twice with TEA-HCl buffer (100 mmol/L, pH 5.0) and used for the reduction activity assay.

### Dry cell weight (DCW) determination

Fermentation samples (50 mL) were centrifuged (8,000 rpm) for 10 min at 4°C. The wet cells from the samples were washed twice with distilled water and dried (105°C, 24 h) to a constant mass. The biomass was expressed as dry cell weight per liter of culture medium; the values given are averages of experiments conducted at least in triplicate. A calibration curve (dry cell weight, g *vs*. OD at 420 nm, *R*^2 ^= 0.9987) was established with experiments. Cell growth was determined by measuring optical density at 420 nm which was then converted to DCW.

### Residual carbon source determination

The residual carbon source concentration was detected using a 3, 5-dinitrosalicylic (DNS) acid method [30].

### Reduction activity assay

4'-Chloroacetophenone reduction was employed as a model reaction for assaying the reduction activity of *Acetobacter *sp. CCTCC M209061. In a typical experiment, 2.0 mL of TEA-HCl buffer (100 mmol/L, pH 5.0) containing 130.6 mmol/L of isopropyl alcohol (the source of reducing equivalents) and 5.0 mmol/L of 4'-chloroacetophenone were added to a 10-mL Erlenmeyer flask capped with a septum, and pre-incubated in a water-bath shaker at 30°C and 160 rpm for 10 min. Then, the bioreduction reaction was initiated by adding the harvested wet cells to the mixture. Aliquots (50 *μ*L) were withdrawn at specified time intervals. The product and the residual substrate were extracted twice with isopropyl ether (100 *μ*L) containing 5.0 mM 3'-methoxyacetophenone (internal standard) prior to GC analysis. The specific reduction activity of the cell was defined as the initial reaction rate per gram of DCW.

The reduction of 4-(trimethylsilyl)-3-butyn-2-one was monitored using the method reported previously [10].

### GC analysis

In the case of 4'-chloroacetophenone reduction, reaction mixtures were assayed with a GC 2010 gas chromatograph (Shimadzu Corp., Kyoto, Japan) equipped with an HP-Chiral column (30 m × 0.25 mm, coating thickness 0.25 *μ*m, J&W Scientific, USA) and a flame ionization detector. The column temperature was held at 140°C for 9 min. Nitrogen was used as the carrier gas at a flow rate of 3 mL/min. The split ratio was 1:100 (v/v). The injector and the detector temperatures were set at 250°C and 250°C, respectively. The retention times for 4'-chloroacetophenone, 3'-methoxyacetophenone, (*R*)-1-(4-chlorophenyl) ethanol and (*S*)-1-(4-chlorophenyl) ethanol were 4.736 min, 6.197 min, 7.596 min and 8.021 min, respectively. The average error for this determination was less than 1%. All reported data were averages of experiments performed at least in duplicate.

For 4-(trimethylsilyl)-3-butyn-2-one reduction, the reaction mixtures were assayed according to the chiral GC analysis method reported previously [31].

### Effect of medium components and culture conditions using the one-factor-at-a-time method

To determine the most important medium components and culture conditions for the growth and reduction activity of *Acetobacter *sp. CCTCC M209061, medium and culture parameters were varied singly. The parameters explored were type of carbon source (glucose, fructose, galactose, arabinose, ribose, xylose, lactose or sucrose; each at a concentration of 10 g/L), nitrogen source (peptone, yeast extract, ammonium sulfate or urea; each at a total nitrogen concentration of 2.15 g/L), mineral element (K_2_HPO_4_, MgSO_4_·7H_2_O, CaCl_2_, ZnSO_4_·7H_2_O, MnSO_4_·H_2_O, FeSO_4 _or CuSO_4_·5H_2_O; each at different concentrations, as detailed in the Results), vitamins (thiamine, nicotinic acid, pyridoxine, ascorbic acid or biotin, each at a concentration of 2 mg/L), and various physical conditions (initial pH, temperature and shaking rate). Samples were withdrawn from each culture at specified time intervals for estimation of biomass via OD_420 nm _and the harvested wet cells were used for reduction activity assays. During the one-factor-at-a-time optimization, the factors (carbon source, nitrogen source, mineral elements, vitamins, pH, temperature and shaking rate) were optimized sequentially in the order described in the Results (sections 3.1-3.6) and the optimized value for each factor was used in subsequent stages of the optimization.

### Optimization of medium components and culture conditions by response surface methodology

As detailed in the Results section, the above one-variable-at-a-time method gave the highest biomass and activity in the presence of glucose, soy peptone, and MnSO_4_·H_2_O, and indicated that the initial pH was important also. The response surface methodology (RSM) was then used to optimize the quantitative concentrations and levels of these variables. The experimental design was performed by using the software Design Expert 7 (Stat-Ease, Minneapolis, MN, USA). A full 2^4 ^factorial central composite rotatable design (CCRD) that contained six replications at the central points (n_0 _= 6) was performed, which meant that a total of 30 experiments were required. The levels of each factor tested in the CCRD experiment are shown in Table 3. Biomass and reduction activity for each shake-flask run were determined after a fermentation period of 30 h, and the observed values were affected by the individual factors and their interactions. The actual concentrations of each medium component were coded to facilitate multiple regression analysis. The second-order polynomial model used to simulate experimental data give the equation:

$$Y_{i}=\beta_{0}+\overset{k}{\underset{i=1}{\sum }}\beta_{i}X_{i}+\overset{k}{\underset{i=1}{\sum }}\beta_{ii}X_{i}^{2}+\overset{k}{\underset{i<j}{\sum }}\sum \beta_{ij}X_{i}X_{j}$$

where *Y*_i _is the predicted response, subscripts i and j take values from 1 to 4, *β*_0 _is a constant, *β*_i _is the linear coefficient, *β*_ii _is the quadratic coefficient, *β*_ij _is the cross-product coefficient, *k *is the number of factors (which was 4 in this study), *X*_i _and *X*_j _are the coded dimensionless values of the investigated variables that influence the response variable *Y*_i_. The analysis of variance (ANOVA) and the graphical analysis of the data were also performed by using the software Design Expert 7 (Stat-Ease, Minneapolis, MN, USA). The statistical significance of the quadratic model was assessed using an *F*-test, and the quality of fit was evaluated by *R*^2^. The significances of the regression coefficients were tested by a t-test, and the *P*-values were used as a tool to check the significance of each coefficient. The optimal levels of the investigated factors needed for simultaneous maximization of two responses (biomass and reduction activity) were established using the overall desirability function, which is the geometric mean of the individual desirability [15].

## Authors' contributions

WYL and MHZ participated in the design of the study; XHC carried out experiments and wrote the draft of manuscript; TJS assisted with data interpretation and revision of the manuscript. All authors read and approved the final manuscript.
